# Chemopreventive Effects of *Peucedanum praeruptorum* DUNN and Its Major Constituents on SGC7901 Gastric Cancer Cells

**DOI:** 10.3390/molecules15118060

**Published:** 2010-11-09

**Authors:** Taigang Liang, Wenyan Yue, Qingshan Li

**Affiliations:** 1 School of Pharmaceutical Science, Shanxi Medical University, No 56, Xinjian Nan Road, Taiyuan 030001, Shanxi, China; E-Mails: ltaigang@gmail.com (T.L.); yuewenyanhappy@163.com (W.Y.); 2 School of Public Health, Shanxi Medical University, No 56, Xinjian Nan Road, Taiyuan 030001, Shanxi, China

**Keywords:** *Peucedanum praeruptorum* DUNN, (±) praeruptorin A, (±) praeruptorin B, chemopreventive effect, SGC7901 gastric cancer cell

## Abstract

In this study, the effects of *Peucedanum praeruptorum* DUNN methanolic extract (PPME) and its major constituents on SGC7901 human gastric cancer cells were evaluated. Two pyranocoumarins, namely, (±) praeruptorin A (PA) and (±) praeruptorin B (PB), were isolated from PPME. A high performance liquid chromatographic (HPLC) method was developed to determine the contents of PA and PB in PPME. The anti-proliferative and cytotoxic actions of PPME were observed using the 3-(4,5-dimethyl-thiazol-2-yl)-2,5-diphenyltetrazolium bromide (MTT) and release of lactate dehydrogenase (LDH) assays. At 300 μg/mL, PPME inhibited cell growth by 51.2% (*P* < 0.01), probably linked to the high concentration of PA and PB. Both PA and PB exhibited antiproliferative and cytotoxic activities on the SGC7901 cells. Furthermore, the active principle compound, PA, also enhanced the actions of doxorubincin (DOX) on SGC7901 cells. Cell growth decreased higher with the combined treatment of PA and DOX than that with the chemotherapy agent applied alone, suggesting that PA could reduce the dose of DOX for the desired effects.

## 1. Introduction

As one of the most common malignancies in the world, gastric cancer is becoming a serious public health problem. The highest incidence rate occurs in Eastern Asia, including China, Japan, Republic of Korea, Democratic Republic of Korea and Mongolia, where the rates are 46 per 100,000 males and 21 per 100,000 females [1]. Chemoprevention, as a therapy method, is applied extensively to deal with the cancers especially in preventing metastasis, which proves to be capable of avoiding one-third of cancer deaths [2]. Howerer, the therapeutic effect of chemotherapy drugs is limited due to their adverse reactions [3,4] and multidrug resistance (MDR) of tumor cells to chemotherapeutic agents [5]. In the last few decades, natural products have become an increasingly important source of potential anticancer agents [6].

*Peucedanum praeruptorum* DUNN (*P. praeruptorum*) is commonly called “Baihua Qianhu”. As a well-known and popular herb drug in Chinese medicine, it has been officially listed in the Pharmacopoeia of the People’s Republic of China [7]. Its roots are credited with latent-heat-clearing, antipyretic, antitussive and mucolytic actions; chiefly, it can be used for treatment of cough with thick sputum and dyspnea, upper respiratory infections, and nonproductive cough [8]. Modern pharmacological studies show that the extract of *P. praeruptorum* has a wide variety of activities, including antibacterial, coronary dilatory [8], antipressure [9], myocardial protection [10], antitumor effects [11] and so on.

Phytochemical investigations reveal that coumarins are widely distributed in this plant [12,13,14,15]. Among them, angular-type pyranocoumarins (seselins) were regarded as the principal components responsible for the main pharmacological activities. Angular–type pyranocoumarins are polyphenolic compounds which occur in an angular form with the pyrano ring attached to the 7,8 position of the benzo-2-pyrene nucleus [16]. The compounds (±) praeruptorin A (PA), (±) praeruptorin B (PB), are characteristic chemical constituents in *P. praeruptorum*. As naturally occurring angular-type pyranocoumarins, PA and PB show a variety of biological activities, including anticancer [17], antileukemia, calcium antagonistic action [18,19,20], and anti-platelet aggregation effects [21]. Furthermore, recent investigation has indicated PA also could suppress P-glycoprotein (P-gp) expression and reverse P-gp mediated MDR in KBV1 cells [14].

To the best of our knowledge, the effects of *P. praeruptorum* and its constituents on gastric cancer cells have not been tested. In this study, PA and PB were isolated from *P. praeruptorum* and their contents in *P. praeruptorum* methanolic extract were determined using a HPLC method. Secondly, the actions of *P. praeruptorum* methanolic extract and two isolated compounds on SGC7901 human gastric cancer cells were investigated. Finally, the effects of PA as a chemoadjuvant for gastric cancer treatment were also evaluated.

## 2. Results and Discussion

### 2.1. Isolation and HPLC analysis of two pyranocoumarins in PPME

**Figure 1:**
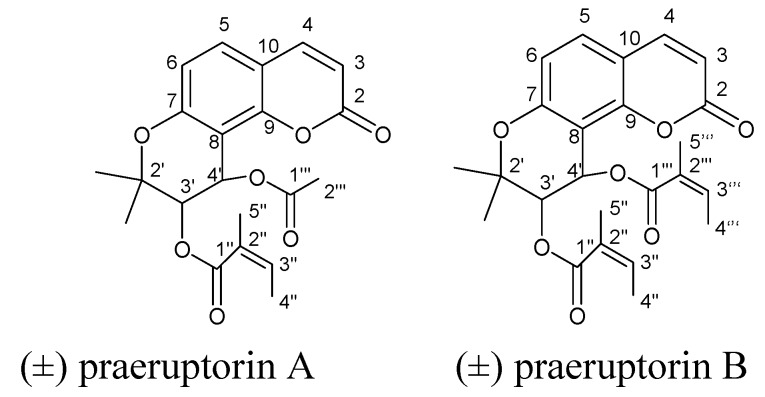
Chemical structures of (±) praeruptorin A and (±) praeruptorin B.

Compounds **1** and **2** were identified by spectroscopic analysis and comparison with literature data [12]. Their spectral data agreed well with those of PA and PB, respectively. The structures are shown in Figure 1.

**Figure 2:**
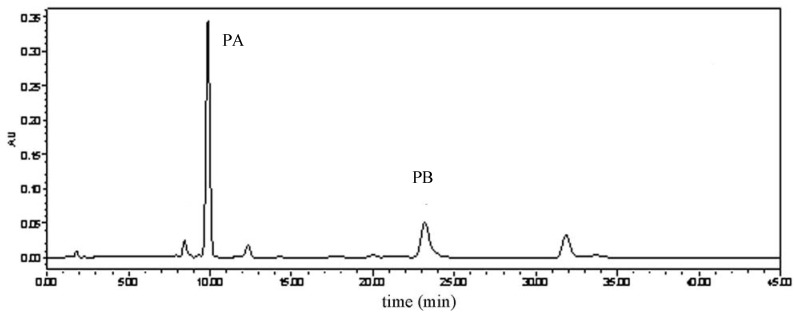
HPLC analysis of *P.praeruptorum* methanolic extract (PPME).

In PPME, PA and PB, were identified by comparison to the retention times of authentic standards analyzed under identical analytical conditions. The HPLC analysis of PPME is shown in Figure 2. The contents of two major coumarins in the extract were PA (53.98%) and PB (26.21%), respectively. This result indicates that PA and PB are the predominant components in *P. praeruptorum* methanolic extract which is consistent with the previous report [25]. It can be concluded that two coumarins, especially PA, were responsible for the pharmacological properties of PPME.

### 2.2. Effects of PPME on SGC7901 cell proliferation

**Figure 3:**
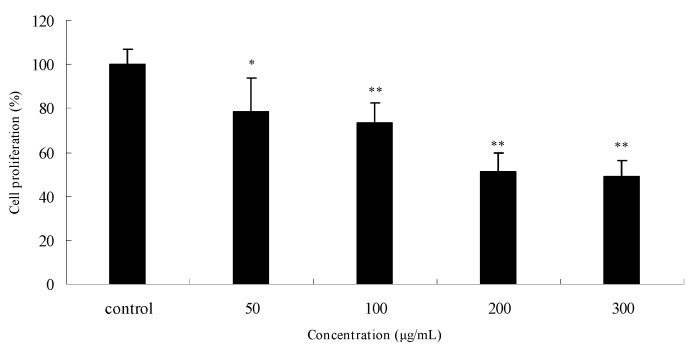
Anti-proliferative effects of *P. praeruptorum* methanolic extract (PPME) on SGC7901 human gastric cancer cells after 24 h treatment.^*^P < 0.05; ^**^P < 0.01 *vs.* control (proliferation as 100%).

The antiproliferative effects of PPME on SGC7901 human gastric cancer cells were evaluated according to the MTT assay results. As shown in Figure 3, PPME significantly inhibited cell proliferation in a concentration-dependent manner after treatment for 24 h. Compared to the control (normalized to 100%), the cell growth was reduced by 21.4% (*P* < 0.05), 25.6% (*P* < 0.01), 48.9% (*P* < 0.01), and 51.2% (*P* < 0.01) at 50, 100, 200 and 300 μg/mL, respectively. Cell proliferation was assessed by MTT conversion into an insoluble formazan dye. This method demonstrates functional mitochondrial dehydrogenases which are present in living cells [26]. The result indicated that PPME exhibits specific anti-proliferation activity towards SGC7901 human gastric cancer cells.

### 2.3. Effects of PPME on cytotoxicity

**Figure 4:**
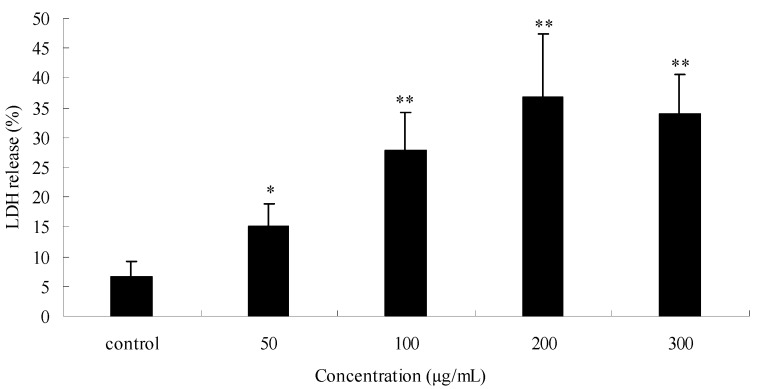
Effects of *P. praeruptorum* methanolic extract (PPME) on SGC7901 human gastric cancer cells cytotoxicity determined by lactate dehydrogenase (LDH) release after 24 h treatment.^*^P < 0.05; ^**^P < 0.01 *vs* control (6.6% LDH release).

The cytotoxicity assay detects the release of stable cytosolic enzyme LDH into the culture medium, due to cell membrane injury [27]. The data are depicted in Figure 4. The figure shows a dose-dependent increase in LDH release at the range of 50-200 μg/mL after exposure PPME for 24 h. The activities of LDH release were 15.0% (P < 0.05), 27.7% (P < 0.01), 36.9% (P < 0.01) and 34.0% (P < 0.01), respectively at the concentrations of 50, 100, 200 and 300 μg/mL, as compared to control cells (6.6% LDH release). The measurement of LDH release is useful in assessing the cytotoxicity of cells [28]. The LDH activity is closely related to the decline in the surviving fraction. The exact mechanism of the cytotoxic effects of PPME is still unknown, and LDH release by PPME could be one of the reasons leading to cell death.

### 2.4. Effects of PA and PB on SGC7901 cell proliferation and cytotoxicity

**Figure 5:**
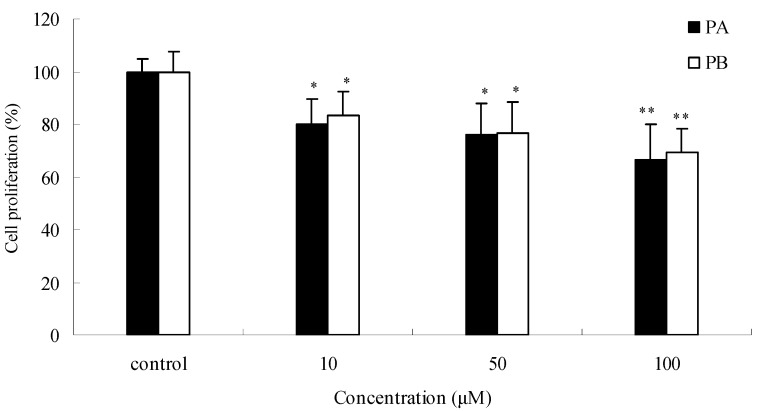
Anti-proliferative effects of (±) praeruptorin A (PA) and (±) praeruptorin B (PB) on SGC7901 human gastric cancer cells after 24 h treatment. ^*^P < 0.05; ^**^P < 0.01 *vs.* control (proliferation as 100%).

**Figure 6:**
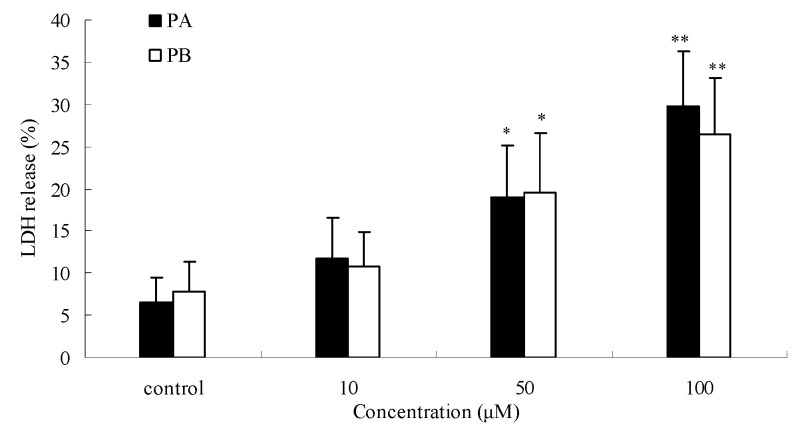
Effects of (±) praeruptorin A (PA) and (±) praeruptorin B (PB) on SGC7901 human gastric cancer cells cytotoxicity determined by lactate dehydrogenase (LDH) release after 24 h treatment.^*^P < 0.05; ^**^P < 0.01 *vs.* control.

To explore the inhibitory activities of single chemical constituents in *P. praeruptorum*, the effects of PA and PB (found in relatively high contents in the root) on SGC7901 cells were determined. As shown in Figure 5, compared to control, PA at concentrations of 10, 50, and 100 μM inhibited SGC7901 cells growth by 19.9%, 24.1%, and 33.7%, respectively (all *P* < 0.05) in a dose-dependent manner. Similar effects were observed with PB. PA and PB also displayed cytotoxicity on SGC7901 cells by LDH release assay (Figure 6). The result indicates LDH activities increased in a concentration-dependent manner in the cell culture supernatant after 24 h of incubation with PA and PB at 10, 50, and 100 μM. The maximum LDH-release were 29.7% for PA (*P* < 0.01), and 26.5% for PB (*P* < 0.01), compared with the control. PA and PB displayed a marked anti-proliferative and cytotoxic effect. The actions of PPME on SGC7901 cells could be attributed to the high contents of PA and PB. 

### 2.5. Effect of PA on the anti-proliferative action of chemotherapeutic agents

**Figure 7:**
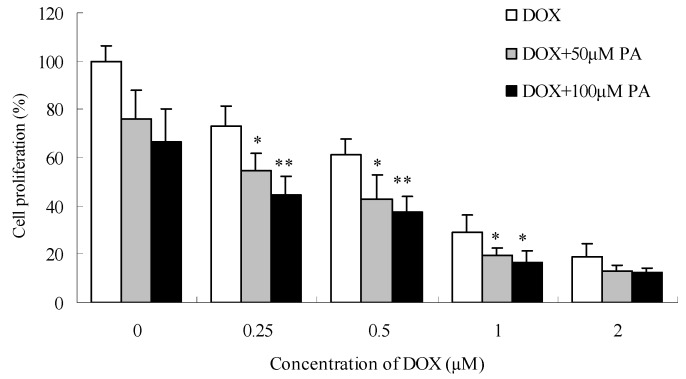
(±) praeruptorin A (PA) enhances the anti-proliferative effects of doxorubicin (DOX) on SGC7901 human gastric cancer cells after 24 h treatment. ^*^P < 0.05; ^**^P < 0.01 *vs.* DOX only.

The cytostatic drug doxorubicin (DOX) is a well-known chemotherapeutic agent which is used in treatment of a wide variety of cancers, including gastric tumour, [29]. The effects of PA, as the main component in PPME, on the anti-proliferative effects of DOX towards SGC7901 cells were investigated. As shown in Figure 7, When SGC7901 cells were treated only with DOX, cell growth decreased by 27.0%, 38.8%, 70.8% and 81.3% at the concentrations of 0.25, 0.5, 1.0 and 2.0 μM, respectively. After PA (50 and 100 μM) were combined with DOX at the above concentrations, the cell growth was decreased further, and PA displayed a concentration-dependent effect on DOX. For example, a combined treatment of DOX at 0.25 and 0.5 μM with PA at 100 μM decreased cell growth by 55.4% and 62.8% (*P* < 0.01 *vs.* DOX alone). The results suggested that the DOX dose could be obviously reduced to achieve a similar effect and the action sensitivity on SGC7901 could be improved after treated with PA.

DOX is a regular chemotherapeutic agent known for its strong cell killing ability. However, DOX treatment is often hampered by severe side effects like heart failure [30] and resistance of cancer cells to therapy [31]. Reduction of its dose and maintenance of its high efficacy will be necessary in the treatment of tumors. More efforts have been made to explore the combination of chemotherapeutic drugs with some other agents.

PA can sensitize SGC7901 cells which may be attributed to its inhibitory effect of P-gp. P-gp, an ATP-dependent drug efflux pump, is extensively distributed and expressed in the cancer cell plasma membrane and extrudes a spectrum of anticancer drugs from cancer cells and decreases effective intracellular drug concentrations [32]. Over-expression of P-gp represents one of the major mechanisms that contribute to multidrug resistance (MDR) in cancer cells [33]. PA was reported to suppress P-gp expression and reverse Pgp-MDR in KBV1 cells [14]. Furthermore, DOX was a classical P-gp substrate [34], PA maybe increase DOX intracellular accumulation by inhibiting Pgp-related drug efflux. In this study, we observed that PA could sensitize SGC7901 cells and reduce the DOX dose.

On the other hand, hepatotoxicity associated with coumarins were reported due to the formation of a coumarin 3,4-epoxide intermediate and leads to increases in plasma ALT and AST in rats [35,36]. Hence, further investigation is required to find out the adverse effects, especially to hepatotoxicity, of PA.

## 3. Experimental

### 3.1. Plant materials

The roots of *P. praeruptorum* were collected from Anhui Province, China, in 2009. The voucher specimen was identified by the Department of Pharmacognosy, Shanxi Medical University, Taiyuan, China. 

### 3.2. Chemicals and reagents

HPLC grade methanol was obtained from Fisher Scientific (Pittsburgh, PA, USA). MilliQ water was supplied by a water purification system (Millipore, Bedford, MA, USA). DOX, 3-(4,5-dimethylthiazol-2-yl)-2,5-diphenyltetrazolium bromide (MTT) and dimethyl sulphoxide (DMSO) were obtained from Sigma (St. Louis, MO, USA). Other chemicals in the studies were of highest quality commercially available from local suppliers in Shanghai, China.

### 3.3. Cell culture

Human gastric cancer cell line, SGC7901, was purchased from the Type Culture Collection of Chinese Academy of Sciences (Shanghai, China). Tumor cells were cultured in RPMI-1640 medium (Gibco, Grand Island, NY, USA) containing 10% fetal bovine serum (Sijiqing, Zhejiang, China), 100 U/mL penicillin, 100 μg/mL streptomycin (Gibco), and 2 mM L-glutamine (Gibco) in an atmosphere of 37 ºC in 5% CO_2_, and the cells were harvested at approximately 80-90% confluency using 0.25% trypsin-EDTA (Gibco). DMSO concentrations in all cell culture experiments did not exceed 0.1%.

### 3.4. Isolation of PA and PB

The roots of *P. praeruptorum* (300 g) were extracted with MeOH (2 × 3 L) under reflux for 2 h. The MeOH extract was filtered and exhaustively concentrated to produce a MeOH extract (49 g). The MeOH extract was partitioned between CHCl_3_ (3 × 1 L) and H_2_O (1 L). The CHCl_3_-soluble fraction (12 g) was chromatographed on a silica gel column (200-250 mesh) with a gradient of EtOAc and petroleum ether (1:10 to 3:7) to afford two subfractions I and II. Subfraction I was further separated by silica gel (250-300 mesh) column chromatography, eluting with EtOAc-petroleum ether (1:9), to yield compound **2** (45 mg). Subfraction II was recrystallized in EtOH to provide compound **1** (310 mg). The structures of compounds were elucidated on the basis of their ^1^H-NMR spectra, recorded on a Bruker Avance 300 MHz NMR spectrometer. The chemical shifts are given in *δ* (ppm) and coupling constants in Hz. Mass spectral were performed on a ABI 3200 mass spectrometer. 

PA (**1**): White amorphous powder; MS (ESI): 409 [M+Na]^+^; ^1^H-NMR (300 MHz, CDCl_3_): 6.24 (1H, d, *J* = 9.6 Hz, 3-H), 7.61 (1H, d, *J* = 9.6 Hz, 4-H), 7.34 (1H, d, *J* = 8.6 Hz, 5-H), 6.81 (1H, d, *J* = 8.6 Hz, 6-H), 5.40 (1H, d, *J* = 5.0 Hz, 3′-H), 6.55 (1H, d, *J* = 5.0 Hz, 4′-H), 1.44 (3H, s, C-2′- CH_3_), 1.38 (3H, s, C-2′- CH_3_), 6.12 (1H, br q, *J* = 7.2 Hz, 3′′-H), 1.96 (3H, br d, *J* = 7.2 Hz, 4′′-H), 1.87 (3H, brs, 5′′-H), 2.10 (3H, s, -COCH_3_); ^13^C-NMR (100 MHz, CDCl_3_): 160.01 (C-2), 113.08 (C-3), 143.32 (C-4), 129.16 (C-5), 114.32 (C-6), 156.71 (C-7), 106.94 (C-8), 153.91 (C-9), 112.50 (C-10), 77.68 (C-2′), 69.68 (C-3′), 60.93 (C-4′), 22.91 (C-2′-CH_3_), 24.88(C-2′-CH_3_), 166.40 (C-1′′), 126.83 (C-2′′), 139.86 (C-3′′), 15.73 (C-4′′), 20.46 (C-5′′), 169.87 (C-1′′′), 20.60 (C-2′′′). 

PB (**2**): White amorphous powder; MS (ESI): 449 [M+Na]^+^; ^1^H-NMR (300 MHz, CDCl_3_): 6.19 (1H, d, *J* = 9.6 Hz, 3-H), 7.55 (1H, d, *J* = 9.6 Hz, 4-H), 7.35 (1H, d, *J* = 8.6 Hz, 5-H), 6.78 (1H, d, *J* = 8.6 Hz, 6-H), 5.40 (1H, d, *J* = 4.8 Hz, 3′-H), 6.64 (1H, d, *J* = 4.8 Hz, 4′-H), 1.42 (3H, s, C-2′-CH_3_), 1.46 (3H, s, C-2′-CH_3_), 6.11 (1H, br q, *J* = 7.2 Hz, 3′′-H), 6.02 (1H, br q, *J* = 6.8 Hz, 3′′′-H), 1.98 (3H, d, *J* = 7.2 Hz, 4′′-H), 1.95 (3H, d, *J*= 6.8 Hz, 4′′′-H), 1.82 (3H, br s, 5′′-H), 1.80 (3H, br s, 5′′′-H); ^13^C-NMR (100 MHz, CDCl_3_): 159.84 (C-2), 113.56 (C-3), 143.32 (C-4), 129.20 (C-5), 114.38 (C-6), 156.70 (C-7), 107.55 (C-8), 154.38 (C-9), 112.47 (C-10), 77.32 (C-2′), 70.15 (C-3′), 70.03 (C-4′), 22.43 (C-2′-CH_3_), 25.31 (C-2′-CH_3_), 166.38 (C-1′′), 127.34 (C-2′′), 139.82 (C-3′′), 15.79 (C-4′′), 20.33 (C-5′′), 166.16 (C-1′′′), 127.07 (C-2′′′), 138.52 (C-3′′′), 15.52 (C-4′′′), 20.35 (C-5′′′). 

### 3.5. Preparation of P. praeruptorum methanolic extract (PPME)

Dried powders of ground material (5 g) were extracted under reflux with methanol (50 mL) at room temperature for 2 h. The extract was filtered through Whatman No.4 filter paper. The residues were re-extracted in the same manner. After that, the two filtrates were combined and evaporated under reduced pressure using a rotary vacuum-evaporator at 50 ºC. The resulting dry materials were collected and stored at -20 ºC in the dark for further analysis. 

### 3.6. High performance liquid chromatography (HPLC) analysis.

The HPLC system was a Waters 2695 instrument (Milford, MA, USA) equipped with a quaternary pump, automatic injector, a photodiode array detector (Model 2696), and Waters Millennium software for peak identification and integration. The separation was carried out on a Zorbax SB-C_18 _column (150 × 4.6 mm, 5.0 µm) with isocractic mobile phase composed of methanol-water (68:32, *v/v*). The flow rate was 0.9 mL/min, the column temperature was maintained at 25 ºC, injection volume was 20 μL, the optimum wavelength was set at 323 nm and UV spectra were recorded in the range 190-400 nm. The standard solutions of PA and PB were prepared at series concentrations to make a calibration curve. Extract sample was dissolved in methanol and filtered through a nylon membrane filter (0.45 μm) before injection. PA and PB were identified by their retention times after comparison with authentic markers and quantified by using an external assay.

### 3.7. Cell proliferation by MTT assay

The cell viability was assessed by the MTT assay, which is based on the reduction of the dye MTT to formazan, an insoluble intracellular blue product, by cellular dehydrogenases [23]. Briefly, SGC7901 cells were seeded in 96-well plate at a density of 1.0 × 10^5^ cells per well and cultured overnight. Following incubation with various concentrations of PPME, PA, PB, DOX and DOX combined with given concentrations of PA for 24 h respectively, then 20 μL of MTT solution (0.5 mg/mL in PBS) was added in each well and the plate was incubated at 37 ºC for 4 h. Subsequently, the formazan was dissolved with 100 μL dimethyl sulphoxide (DMSO) after the medium was removed. Finally, the absorbency of each well was measured at 570 nm using a microplate reader (BioRad, Model 680, USA), and the percentage viability was calculated. The viability of the control cells, from the untreated cultures, was defined as 100%. 

### 3.8. Cytotoxicity by LDH release assay

Cytotoxicity was determined by measuring the release of lactate dehydrogenase (LDH) [24]. The SGC7901 cells were incubated with various concentrations of PPME, PA, and PB for 24 h, respectively. The medium was then collected and the cells were lysed with 1% Triton X-100 for 5 min. The LDH activity was measured using a commercial LDH assay kit (Jiancheng Corporation, Nanjing, China) following the manufacturer’s instructions. The amount of LDH leakage was calculated from the ratio of LDH activity in the medium to the sum of the LDH activity in the medium and in the cell lysate.

### 3.9. Statistical analysis

Results are presented as mean±standard error (SE). Data were analyzed using Student's t-test and analysis of variance (ANOVA) for repeated measures. The level of statistical significance was set at *P* < 0.05.

## 4. Conclusions

In this study, *P. praeruptorum* methanolic extract (PPME) has shown definite anti-proliferative and cytotoxic action on SGC7901 cells. Its pharmacological activities could be attributed to its two major constituents, PA and PB, which were isolated from PPME and determined by HPLC. Both PA and PB exhibited marked antiproliferative and cytotoxic activities on the SGC7901 cells. Furthermore, the principle component, PA, also enhanced the actions of DOX on SGC7901 cells. The combined treatment of PA and DOX can sensitize SGC7901 cells and reduce the dose of DOX to achieve the desired effects.
